# Protein Kinases and Parkinson’s Disease

**DOI:** 10.3390/ijms17091585

**Published:** 2016-09-20

**Authors:** Syed Jafar Mehdi, Hector Rosas-Hernandez, Elvis Cuevas, Susan M. Lantz, Steven W. Barger, Sumit Sarkar, Merle G. Paule, Syed F. Ali, Syed Z. Imam

**Affiliations:** 1 Department of Geriatrics, University of Arkansas for Medical Sciences, Little Rock, AR 72205, USA; SJMehdi@uams.edu (S.J.M.); BargerStevenW@uams.edu (S.W.B.); 2 Division of Neurotoxicology, National Center for Toxicological Research/US Food and Drug Administration, Jefferson, AR 72079, USA; Hector.Rosas-Hernandez@fda.hhs.gov (H.R.-H.); Elvis-Yane.Cuevas-Martinez@fda.hhs.gov (E.C.); Susan.Lantz@fda.hhs.gov (S.M.L.); Sumit.Sarkar@fda.hhs.gov (S.S.); Merle.Paule@fda.hhs.gov (M.G.P.); Syed.Ali@fda.hhs.gov (S.F.A.); 3 Geriatric Research Education and Clinical Center, Central Arkansas Veterans Healthcare System, Little Rock, AR 72205, USA

**Keywords:** Parkinson’s disease, dopamine, tyrosine kinase, serine/threonine kinase, kinase inhibitors

## Abstract

Currently, the lack of new drug candidates for the treatment of major neurological disorders such as Parkinson’s disease has intensified the search for drugs that can be repurposed or repositioned for such treatment. Typically, the search focuses on drugs that have been approved and are used clinically for other indications. Kinase inhibitors represent a family of popular molecules for the treatment and prevention of various cancers, and have emerged as strong candidates for such repurposing because numerous serine/threonine and tyrosine kinases have been implicated in the pathobiology of Parkinson’s disease. This review focuses on various kinase-dependent pathways associated with the expression of Parkinson’s disease pathology, and evaluates how inhibitors of these pathways might play a major role as effective therapeutic molecules.

## 1. Introduction

Protein phosphorylation is one of the major mechanisms that regulates nearly all eukaryotic cellular activities. It is a reversible and dynamic process that is mediated by protein kinases and phosphoprotein phosphatases. The functions of protein kinases and their phosphatase counterparts are evolutionarily conserved from prokaryotic (e.g., *Escherichia coli*) to multicellular eukaryotic organisms (including *Homo sapiens*) [1]. Phosphorylation regulates virtually every fundamental process of living beings, including cellular proliferation, differentiation, migration, metabolism, and anti-apoptotic signaling [2]. A kinase catalyzes the transfer of the gamma phosphate from nucleotide triphosphates (usually ATP) to an amino acid side chain of a protein substrate, resulting in an electrostatic and steric change that alters protein function, often through a conformational rearrangement. This functional change of the target protein can include enzymatic activity, cellular location, or its association with other proteins. Therefore, there is a growing interest in the pursuit of drugs that manipulate phosphorylation status in a specific way for the treatment of a number of diseases [3]. A very recent example of such a drug based on our findings of tyrosine kinase involvement in Parkinson’s disease (PD) [4] is the current open-label clinical trial of Nilotinib for Parkinson’s disease [5].

PD is the second most common neurodegenerative disease, and affects up to 5% of individuals over the age of 85 [6]. PD is a movement disorder which is characterized by the clinical symptoms of resting tremor, rigidity, and bradykinesia. These physical symptoms of PD are mainly neuropathologically correlated with the loss of dopaminergic neurons in the substantia nigra (SN) and deposition of Lewy bodies (LB); cytoplasmic aggregates of the protein α-synuclein (α-syn) in this and other brain regions. The movement disorder in PD arises from reduced dopaminergic input to the striatum as a result of nigral degeneration. The current clinical treatments for PD mainly focus on suppressing disease symptoms, but do not restrict disease progression [7]. Therefore, a disease-modifying therapy is an important unmet need in the PD field. Hence, kinases are now being considered as an ideal category of drug targets for PD therapy.

Complex molecular mechanisms underlying the pathogenesis of PD are gradually being elucidated. Research has strongly implicated the dysfunction of kinase activities and phosphorylation pathways in the pathogenesis of PD. Major protein kinases associated with increased risk and cause of PD are PTEN (phosphatase and tensin homolog)-induced putative kinase 1 (PINK1) and leucine-rich repeat kinase 2 (LRRK2). PINK1 and LRRK2—along with their associated protein kinase B (AKT) and c-Jun N-terminal kinase (JNK) signaling pathways—are being intensively studied with respect to their relationship to PD. It is believed that deep evaluation of these signaling pathways will reveal potential therapeutic targets for the attenuation of the cardinal symptoms and motor complications in patients with PD in the future.

## 2. Leucine-Rich Repeat Kinase 2 (LRRK2)

Mutations in the *LRRK2* gene are thought to be a common cause of PD [8,9]. LRRK2 is a 286 kDa large multi-domain protein. It encodes both protein kinase and GTPase domains. The majority of pathogenic mutations in LRRK2 lie in its catalytic domains. Various mutations identified in the *LRRK2* gene are found to be associated with the familial forms of PD; polymorphisms in *LRRK2* also contribute to typical idiopathic late-onset PD [10,11,12,13,14]. Collectively, these LRRK2 mutations account for a large portion of autosomal-dominantly inherited PD [15]. LRRK2-associated PD is largely indistinguishable from sporadic PD both clinically and pathologically, which suggests a role for LRRK2 in all forms of PD.

The most common LRRK2 mutation results in the substitution of serine for glycine in the activation loop of the protein kinase domain (i.e., G2019S). This is associated with increased kinase activity and neurotoxicity [16,17]. Some other common mutations include the replacement of arginine by histidine (R1441H), cysteine (R1441C), or glycine (R1441G) in the GTPase domain. These mutations also appear to increase the kinase activity by trapping LRRK2 in a GTP-bound active state [18,19]. Previous studies have already shown that a complex relationship exists between GTP and LRRK2, ultimately demonstrating that GTP binding is required for LRRK2 kinase activity [20,21].

Several studies have sought to determine whether LRRK2 mutations present in PD alter its kinase activity. It is clear that the G2019S mutation significantly increases LRRK2 kinase function and manifests as either autophosphorylation or phosphorylation of generic substrates [16,17,22,23,24,25]. However, mutations in the GTP-binding domain reduce the rate of GTP hydrolysis compared to wild-type LRRK2, suggesting that these mutations indirectly affect kinase activity [26,27]. In cell lines and primary neurons, LRRK2 mutations present in PD patients show enhanced toxicity that result in significantly increased cell death relative to cells with the wild-type protein. Most of the mutations associated with PD appear to cause cell death by altering the features of LRRK2 biology in a way that nonetheless preserves basal kinase function. LRRK2 is a signaling molecule, and its kinase activity is one key part of the signaling process. LRRK2 becomes pathogenic when the kinase is hyperactive or mis-regulated, and this may involve other elements of its signaling pathway. It has also been suggested that LRRK2 or homologs in other species have roles in neurite outgrowth and sorting of molecules along axons [25,28]. Therefore, LRRK2 probably has activities that are important (perhaps even required) for normal neuronal function. Inhibitors of LRRK2 such as GW5074 and sorafenib have been proven to be protective against LRRK2 toxicity using in vitro and in vivo models of PD [29], which may lead the way to clinical studies using specific inhibitors for this kinase.

## 3. Phosphatase and Tensin Homolog (PTEN)-Induced Putative Kinase 1 (PINK1)

PINK1 mutations are thought to be the second-most common cause of recessive PD and are associated with about 1%–8% of familial juvenile PD [30,31]. About 50 missense mutations have been identified in the PINK1 protein [30]. Unlike LRRK2, PINK1 mutations reduce kinase activity and are associated with an atypical form of PD characterized by an early age of onset and slower clinical progression [32,33].

PINK1 contains a serine/threonine protein kinase domain [31] preceded by an N-terminal mitochondrial-targeting motif with a transmembrane domain located between the two. Although mutations have been found throughout the protein, missense mutations—both truncating and destabilizing—are commonly found in the kinase region. These types of mutations support the concept that disease can be caused by mutations that result in loss of function(s). Additionally, research has indicated that overexpression of PINK1 protects cells against both oxidative and apoptotic stressors in a kinase-dependent fashion [34,35]. Furthermore, loss of PINK1 function has been shown to induce oxidative stress [36]. Thus, loss of PINK1 function appears to promote PD-related neurodegeneration.

PD has long been associated with mitochondrial dysfunction, and PINK1 plays an essential role in the maintenance of a healthy population of mitochondria. The kinase is typically trafficked to the inner mitochondrial membrane if that membrane has a strong proton gradient; this results in PINK1 degradation. By contrast, depolarized mitochondria attract PINK1 only to the outer mitochondrial membrane, where it persists and is autophosphorylated. The autophosphorylation of PINK1 helps to recruit parkin to depolarized mitochondria [37], where it is phosphorylated and activated by PINK1 [38,39]. Parkin is a ubiquitin E3 ligase that breaks down dysfunctional mitochondria (mitochondrial autophagy or mitophagy). PINK1 is largely localized to mitochondria, with few changes in concentration or localization seen in the brain of patients with idiopathic PD, although PINK1 immunoreactivity is detected in ~10% of brainstem Lewy bodies (LB) [40]. A case report has described the neuropathology of an early-onset case of PD associated with a homozygous mutation of PINK1. This patient displayed a pattern of LB pathology with atypical Braak LB staging due to the absence of LB. This sheds some light on the reason for longer disease duration of of PINK1-associated PD [41]. Together, these studies indicate that rather than changes in protein levels of PINK1, it is PINK1 function that contributes to PD, as indicated by the majority of PINK1 mutations resulting in a loss of kinase activity [33]. It would indeed be interesting to explore whether PINK1 autophosphorylation or PINK1-induced parkin phosphorylation is altered in sporadic PD, and how this correlates with mitochondrial health and/or neuronal loss. In a zebrafish model of PD, 1-methyl-4-phenyl-1,2,3,6-tetrahydropyridine (MPTP) administration inhibited mitochondrial complex I and blocked PINK1 function, leading to the accumulation of damaged mitochondria and the development of motor impairment symptoms in the embryos. Surprisingly, the administration of melatonin prevented and reverted the Parkinsonian symptoms and restored the PINK1 gene expression and function [42].

## 4. c-Jun N-Terminal Kinase Signaling Pathway (JNK)

JNK is one of the three branches of the mitogen-activated protein kinase (MAPK) superfamily of serine/threonine protein kinases. Other members of this superfamily include the p38 kinases and the extracellular signal-related kinases (ERKs) [43]. In mammals, there are three JNK genes—*Jnk1*, *Jnk2*, and *Jnk3*—on three different chromosomes, and each mammalian *Jnk* gene has alternative splicing forms [44]. JNK is involved in many physiological and pathological processes. The JNK pathway plays a major role in apoptosis; JNK is required for the neuronal cell death induced by many apoptotic stimuli, such as DNA damage, oxidative stress, amyloid β-peptide, tumor necrosis factor, low potassium, excitotoxic stress, 6-hydroxydopamine (6-OHDA), ultra violet irradiation, and deprivation of trophic support [45,46,47,48,49,50,51,52,53,54].

JNK is activated by a number of factors implicated in PD, such as toxicants, inflammatory agonists, and the unfolded protein stress response in endoplasmic reticulum (ER). Previous studies have demonstrated that JNK is significantly activated in several common models of PD induced by toxicants, such as MPTP, 6-OHDA, and lipopolysaccharide (LPS) [55,56,57]. Genetic deletion of JNK2 and JNK3 protect against MPTP-induced neurodegeneration in mice [58]. Kinase inhibitors of JNK have shown neuroprotective effects in the MPTP and 6-OHDA PD models [59,60,61,62,63]. Moreover, studies have shown that antioxidant and anti-inflammatory compounds provide neuroprotection in MPTP and 6-OHDA PD models, at least in part via the inhibition of JNK activation [57,64,65,66].

**Figure 1:**
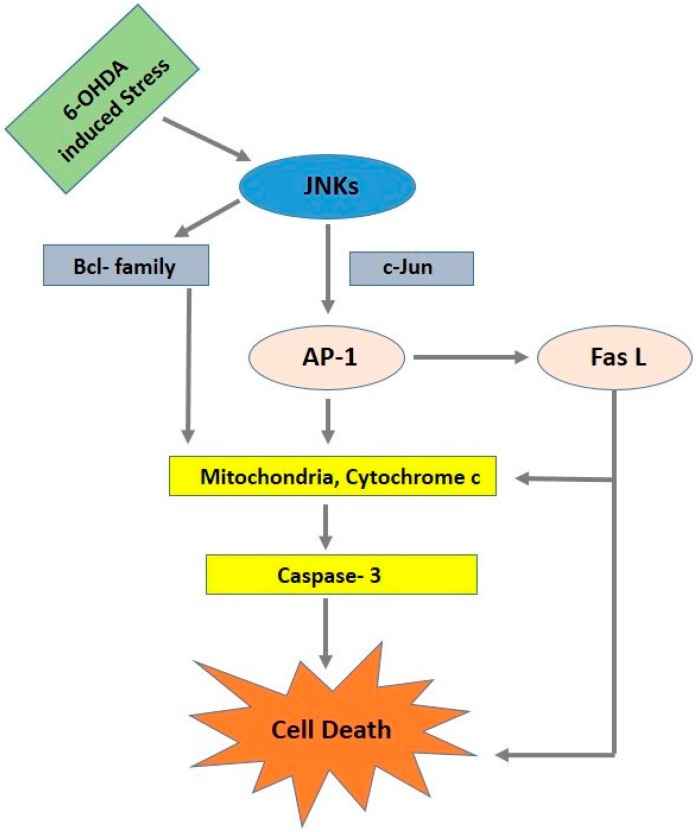
In response to various stressors, c-Jun N-terminal kinase (JNK) is activated and phosphorylates c-Jun, which increases the activity of activating protein-1 (AP-1). AP-1 modulates the transcription genes such as *Fas ligand* (*FasL*) to induce apoptosis via an extrinsic pathway. JNK also appears to activate non-nuclear substrates (such as Bcl-2 family members) to promote cell death via an intrinsic pathway. 6-OHDA: 6-hydroxydopamine.

JNK regulates the activation of downstream pathways—both nuclear and non-nuclear—with activated JNK regulating nuclear substrates like c-Jun. c-Jun plays an important role in neuronal death under in vitro and in vivo conditions [67]. When phosphorylated by JNK, c-Jun participates in the activation of a dimeric transcription factor known as activating protein-1 (AP-1). AP-1 modulates the transcription of a number of genes, such as Fas ligand (*FasL*) [68], *dp5* [69], and *Bim* [70], all of which may contribute to JNK-dependent neuronal death (Figure 1). Apart from the nuclear pathway, JNK also regulates the activation of non-nuclear substrates, and thereby promotes cell death. In 6-OHDA PD models, JNK3 enhances the phosphorylation of 14-3-3 proteins and promotes Bax translocation to the mitochondria. It causes the release of cytochrome *c* and increases caspase-3 activation [71,72].

## 5. AKT (Protein Kinase B) Signaling Pathway

AKT is a serine/threonine protein kinase that regulates multiple cellular processes, such as cell survival, proliferation, and insulin-dependent metabolism. AKT is also known as protein kinase B (PKB). It contains at least three subunits (AKT1/PKBα, AKT2/PKBβ, and AKT3/PKBγ) in humans [73,74]. Research has shown that AKT executes numerous tasks through the phosphorylation of several cellular substrates and plays an important role in physiological and pathological settings. The phosphorylation of AKT/PKB Ser473 is reduced in both the cytosolic and membrane fractions of PD midbrain homogenates [75]. AKT/PKB and its phosphorylation at Ser473 are also consistently reduced in dopaminergic neurons of the SN. Others have confirmed reduced phosphorylation of AKT/PKB at both Thr308 and Ser473 in SN dopaminergic neurons in PD patients [76]. Taken together, research suggests that activation of AKT/PKB is limited to dopaminergic neurons, as non-neuromelanin-containing midbrain neurons express similar levels of both AKT/PKB and phosphorylated AKT/PKB in controls and PD patients.

Defective phosphoinositide (PI) 3-kinase-AKT (PKB) signaling is associated with loss of dopaminergic neurons in PD [75]. Some studies have also shown a significant decrease in the expression of AKT and phospho-Ser473 AKT in tyrosine hydroxylase (TH) immunoreactive dopaminergic neurons in the brains of PD patients [75]. A significant reduction in AKT/PKB Thr308 and Ser473 phosphorylation was observed in 6-OHDA-treated cell-cultures and rodent PD models; this was associated with a marked loss of AKT/PKB activity. Research has also demonstrated that several compounds that stimulate AKT/PKB activity are neuroprotective in the 6-OHDA-administered PD model, as well as other toxicant-induced PD models, including those caused by MPTP and rotenone [77]. Over-expression of *AKT*/*PKB* in rodent brain also protects dopaminergic neurons from 6-OHDA-induced cell death [78].

The PI3K/AKT and JNK pathways are the prominent serine/threonine signaling pathways involved in PD. These two pathways determine the equilibrium between neuronal death and survival. AKT/PKB prevents cell apoptosis and promotes cell survival. Studies show that AKT/PKB negatively regulates the phosphorylation and activation of JNK by directly phosphorylating apoptosis signal-regulating kinase 1 (ASK1) and JNK-interacting protein 1 (JIP1) [79,80]. Therefore, in PD research it is believed that cell death versus cell survival is determined by balancing the activation of the AKT pathway and inhibition of the JNK pathway.

## 6. c-Abl

**Figure 2:**
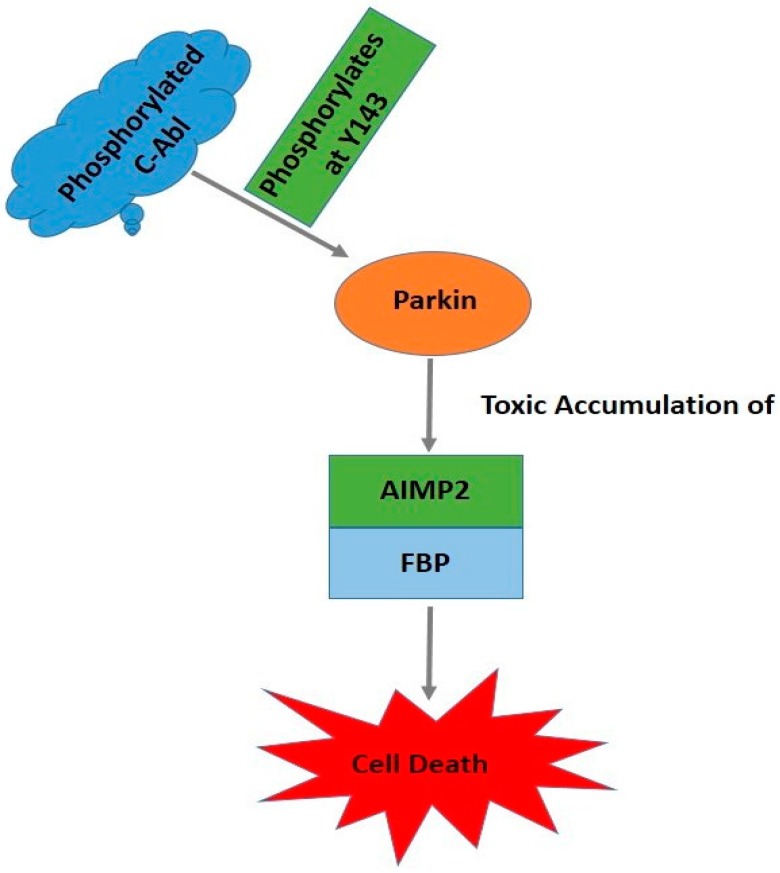
Phosphorylated c-Abl tyrosine phosphorylates parkin at Y143, which leads to the loss of ubiquitin-ligase activity, the accumulation of toxic parkin substrates, and neuronal death. AIMP2: aminoacyl tRNA synthetase complex-interacting multifunctional protein 2; FBP: far up stream element binding protein.

The tyrosine kinase c-Abl regulates several cellular processes that may be linked to PD. c-Abl impacts the development of the central nervous system (CNS) by affecting neurogenesis, neurite outgrowth, and neuronal plasticity [81]. c-Abl is a 120 kDa protein belonging to the cytoplasmic tyrosine-kinase family. Similar to the Src kinases, it possesses sequential SH3 and SH2 domains followed by a core catalytic domain with tyrosine-kinase activity [82,83]. It has been demonstrated that c-Abl has a unique myristoylated N-terminal region that negatively regulates its kinase activity [82]. Several studies have shown the involvement of c-Abl in neurodegenerative diseases such as PD using various experimental model systems [4,84]. Studies show that c-Abl protein level is elevated in the postmortem striatum of PD patients [85]. That study also reinforces a report of increased c-Abl phosphorylation at Y412 in the SN [84,85] and striatum [84] of PD patients. In the earlier study, c-Abl was found to phosphorylate parkin and impair its E3 ligase activity, leading to the loss of dopaminergic neurons in the SN [84] (Figure 2) [4]. More recently, Hebron et al. [85] showed that c-Abl also regulates the clearance of α-syn.

The effects of inhibitors of c-Abl activation (phosphorylation) have been studied in animal models of PD. The brain-permeable second-generation c-Abl tyrosine-kinase inhibitor Nilotinib proved effective in a mice model of PD induced by MPTP. MPTP increased c-Abl phosphorylation, decreased dopamine (DA) levels in the striatum as well as the expression of DA transporter (DAT), and decreased the number of TH-positive neurons in the SN. Administration of Nilotinib 7 days before MPTP decreased the MPTP-induced c-Abl phosphorylation, partially restored the levels of DAT, the DA production in the striatum, and the expression of TH in the SN, suggesting that c-Abl inhibitors may be useful as treatments for PD [86].

To date, there is only one clinical trial for the treatment of PD using Nilotinib [5]. In patients with PD, administration of Nilotinib once a day for 24 weeks was safe and well-tolerated. Nilotinib crosses the blood-brain barrier (BBB), since it was detected in cerebrospinal fluid (CSF) two hours after its administration and succesfuly inhibits c-Abl phosphorylation in the CNS, detected by the ratio of pan-tyrosine Abl relative to total Abl in the CSF. Regarding PD biomarkers, levels of the DA metabolite homovanillic acid (HVA) were increased in the CSF as early as 2 months and remained elevated for up to 6 months. Even when CSF levels of α-synuclein did not seem to significantly change with the drug treatment, plasma levels of α-syn were elevated after 6 months compared to 2 months, and in some subjects remained elevated at month 9—3 months after the end of the treatment—suggesting that Nilotinib may stabilize α-syn levels. All of these molecular changes induced by Nilotinib were related with improvement in motor and non-motor symptoms of PD, suggesting a benefical effect of the drug in the treatment of PD. Although the sample size in this study was small and more conclusive trials need to be conducted, these studies suggest that c-Abl inhibitors can be succesfully used in the treatment of PD [5].

## 7. Concluding Remarks

Current therapies for PD only succeed in managing symptoms for a relatively short period of time and fail completely to impact the progression of the disease. The prevalence, morbidity, and mortality of age-related neurological disorders such as PD increase dramatically with age, and the ongoing expansion of the elderly population signals major increases in impact. Although no serine/threonine kinase inhibitors have yet made it to the clinic as therapeutics for PD, various inhibitors are either in clinical trials or in the pipeline. To date, no known inhibitors of PINK1 or AKT have shown any signs of neuroprotection in a PD model, but a special inhibitor of JNK, SP600125, has been shown to protect dopaminergic neurons in a sub-acute MPTP model of PD [87]. On the other hand, LRRK2 inhibitors have made significant advances as potential therapeutic targets for PD [29,88]. A recent study has shown that a selective LRRK2 inhibitor can be well tolerated in rats and reduces α-syn-induced neurodegeneration [89]. Furthermore, a LRRK2 GTP-binding inhibitor has been shown to reduce neurodegeneration [90]. Finally, sunitinib—a multi-kinase inhibitor that also inhibits LRRK2 kinase—is in clinical trial in order to establish an on-target assay for LRRK2 inhibitors as a therapeutic potential in PD (NCT01860118). With regards to tyrosine kinase inhibitors, neuronal death mechanisms mediated by activated c-Abl seem to provide a very effective therapeutic target. Various c-Abl inhibitors have been shown to provide neuroprotection against dopaminergic damage in different cellular and animal models of PD [4,84,85,86,91]. A recent study has also shown that inhibition of c-Abl improves motor behavior in a mouse model of PD [92]. Tyrosine-kinase inhibitors—many of which are already being used clinically for other indications—can be repurposed for the amelioration of cellular injury in age-related neurological disorders. The use of these compounds would provide new therapeutic approaches that might be broadly applicable to age-related neurological disorders.
